# Molecular mechanism of salinity and waterlogging tolerance in mangrove *Kandelia obovata*

**DOI:** 10.3389/fpls.2024.1354249

**Published:** 2024-02-07

**Authors:** Huizi Liu, Xia An, Xing Liu, Sheng Yang, Yu Liu, Xin Wei, Xiaowen Li, Qiuxia Chen, Jinwang Wang

**Affiliations:** ^1^ Zhejiang Institute of Subtropical Crops, Zhejiang Academy of Agricultural Sciences, Wenzhou, China; ^2^ Zhejiang Xiaoshan Institute of Cotton and Bast Fiber Crops, Zhejiang Institute of Landscape Plants and Flowers, Zhejiang Academy of Agricultural Sciences, Hangzhou, China

**Keywords:** *Kandelia obovata*, salt stress, waterlogging stress, transcriptomic analysis, weighted gene co-expression network

## Abstract

Mangrove forests are colloquially referred to as “Earth’s kidneys” and serve many important ecological and commercial functions. Salinity and waterlogging stress are the most important abiotic stressors restricting the growth and development of mangroves. *Kandelia obovata (K. obovata)* is the greatest latitudinally-distributed salt mangrove species in China.Here, morphology and transcriptomics were used to study the response of *K. obovata* to salt and waterlogging stress. In addition, weighted gene co-expression network analysis of the combined gene expression and phenotypic datasets was used to identify core salinity- and waterlogging-responsive modules. In this study, we observed that both high salinity and waterlogging significantly inhibited growth and development in *K. obovata. *Notably, growth was negatively correlated with salt concentration and positively correlated with waterlogging duration, and high salinity was significantly more inhibitive than waterlogging. A total of 7, 591 salt-responsive and 228 waterlogging-responsive differentially expressed genes were identified by RNA sequencing. Long-term salt stress was highly correlated with the measured physiological parameters while long-term waterlogging was poorly correlated with these traits. At the same time, 45 salinity-responsive and 16 waterlogging-responsive core genes were identified. All 61 core genes were mainly involved in metabolic and biosynthesis of secondary metabolites pathways. This study provides valuable insight into the molecular mechanisms of salinity and waterlogging tolerance in *K. obovata,* as well as a useful genetic resource for the improvement of mangrove stress tolerance using molecular breeding techniques.

## Introduction

Mangroves are unique bionetworks of halophytic trees, shrubs, and other woody plants, which develop in tidal zones along tropical and subtropical coastlines (Parida and Jha, 2010; Bai et al., 2021). Mangroves thrive in inhospitable environments and serve unique ecological functions such as purifying water, protecting coastlines, and providing breeding habitats, and they are considered to be one of the most important blue carbon ecosystems (Costanza et al., 1997; Duke et al., 2007; Parida and Jha, 2010; Donato et al., 2011; Alongi, 2014; Lee et al., 2014; Lovelock and Duarte, 2019). Mangrove dispersal is affected by temperature and ocean currents at the global and regional scales, and salinity and flooding duration at the estuarine and intertidal scales (Srikanth et al., 2016). Salinity and flooding are the primary abiotic stressors restricting the growth and development of mangroves. However, our understanding of how mangrove plants respond to harsh environmental conditions at the molecular level is incomplete.

Flooding is often an accompaniment to natural disasters, and the resulting waterlogging of the soil creates a low-oxygen environment that affects plant growth and development (Loreti et al., 2016; Klumb et al., 2017). Mangrove plants have evolved several adaptations to flooding stress, including the development of various types of air roots such as pillar roots, geniculate roots, surface roots, plate roots, and respiratory roots (McKee, 1993; Alongi, 2009; Srikanth et al., 2016). During the formation of air roots, the parenchyma cells gradually disintegrate as the air cavity is enlarged, and the degree of aerated tissue development is positively correlated with the ability to withstand flooding (Curran et al., 1996; Allaway et al., 2001; Chen et al., 2004; Srikanth et al., 2016). Additionally, mangroves and other plants exhibit an array of physiological and molecular responses to flooding stress. In the mangrove *Sonneratia apetala*, long-term waterlogging results in increased root porosity, cell wall lignification, and outer cortex iron plaque content (Cheng et al., 2015). In soybean seedlings, waterlogging decreases protein biosynthesis and lignin deposition in root and hypocotyl cell walls (Komatsu et al., 2010). In poplar, flooding downregulates the expression of 4CL, C4H, CCoAOMT, COMT, F5H, and PAL (Kreuzwieser et al., 2009). In addition, several families of transcription factors (TFs) play important roles in the flooding stress response, including NAC, MYB, and WRKY (Li et al., 2017; Dhungana et al., 2021; Yao, 2021). In rice, overexpression of *OsMYB2* reduces flooding-induced reactive oxygen species (ROS) accumulation, while overexpression of *OsDREB6*, *OsEREBP1*, *OsSNORKEL1*, and *OsSNORKEL2* promotes adaptation to waterlogging (Hattori et al., 2009; Yang et al., 2012; Ke et al., 2014; Jisha et al., 2015).

Salt stress negatively affects all aspects of plant growth and development, as well as the productivity of agroforestry operations and the quality of their products (Safdar et al., 2019; Vishal et al., 2019; Yaldiz and Camlica, 2021). Salt stress can depress growth rates and even lead to the cessation of growth altogether, as seen in rice (Hussain et al., 2017). While well known for providing mechanical support and transporting nutrients and water, roots also rapidly sense and respond to abiotic stressors such as high salinity, waterlogging, and drought (Overvoorde et al., 2010; Petricka et al., 2012; Czyzewicz et al., 2015; Jourquin et al., 2020). Notably, low concentrations of salt promote root growth in the mangroves *Bruguiera sexangula* var. *rhynchopetala* and *Kandelia obovata*, while high concentrations inhibit root growth (Shiau et al., 2017). In addition, salt stress can result in increased cork formation and root vacuole volume in mangroves (Werner and Stelzer, 1990; Parida and Jha, 2010). Woody plants have evolved an array of physiological and molecular mechanisms to adjust their growth and development under adverse conditions. One such mechanism involves the activation of salinity-responsive TFs, such as MYB, bZIP, and GRF (Kim et al., 2004; Ye et al., 2013; Jiang et al., 2018; Nguyen and Cheong, 2018; Wang et al., 2021; Li et al., 2023). Other salinity-responsive TFs have been reported in *Oryza sativa*, *Nicotiana tabacum*, *Solanum lycopersicum*, *Malus domestica*, *Manihot esculenta*, *Eucalyptus camaldulensis*, and *Betula platyphylla* (Liu et al., 2012; Wang et al., 2012; Zhu et al., 2018; Chen et al., 2019; Tran et al., 2019; Zou et al., 2008; Liu et al., 2024).

*K. obovata* is the most latitudinally distributed salt-excreting mangrove plant species in the Northern Hemisphere and has been widely used in coastal wetland ecological restoration (Zhao et al., 2021). Previous studies primarily focused on the changes in morphological structure, accumulation and migration of mineral ions, physiological characteristics, and gene level of *K. obovata* under a single stress factor (Krauss et al., 2008; Liu et al., 2023). In this study, we evaluated the structural and morphological characteristics of *K. obovata* roots under multiple stress factors, including high salinity and waterlogging stress. In addition, we performed RNA sequencing (RNA-seq) and weighted gene co-expression network analysis (WGCNA) to correlate the phenotypic data with gene expression patterns. We extracted gene modules directly related to salt and waterlogging stress and then constructed co-expression networks to discover core TFs and hub genes. We preliminarily identified the regulatory pathway where the core genes are located. This is the first study to identify the key gene network responsive to high salinity and flooding in *K. obovata*. The results presented here will provide a theoretical basis for future studies of the molecular mechanisms underlying the plant abiotic stress response.

## Materials and methods

### Plant materials and stress treatment

All experiments were performed using *K. obovata* hypocotyls collected from the germplasm resource garden located along the Aojiang River (China). All hypocotyl samples were free of pests and diseases, well-developed, and of similar age. The hypocotyls were planted at a depth of 10 cm in a simulation trough filled with silt from the Aojiang River to a depth of 30 cm and cultivated at 21°C–25°C. Experimental plants were subjected to high salinity and waterlogging stress for 6 months prior to analysis. Experimental *K. obovata* plants were subjected to up to 6 months of stress treatment, including high salinity (NaCl; 10‰, 20‰, and 30‰), waterlogging (WL; 3 h, 6 h, and 9 h of flooding), and high salinity–waterlogging (NaCl + WL). Details of the stress tests are shown in **Supplementary Table 1**. Control plants were not subjected to any stressors. Following treatment, the survival rate was calculated.

### Morphological analysis

After 6 months of stress treatment, the plants were carefully harvested to avoid root damage and washed. Leaf and root growth was evaluated using a LA2400 scanner and analyzed using WinRHIZO (Regent Instruments, Québec, QC, Canada). Stem length was measured using a meter stick and averaged. Electronic weighing scales were used to measure the fresh weights of whole plants, roots, stems, leaves, and hypocotyls. To measure the dry weight, whole plants, roots, stems, leaves, and hypocotyls were separately dried to a constant weight in a 60°C forced-air oven.

### RNA extraction and sequencing

Using the RNA extracted from root tissues, RNA-seq was performed to identify genes responsive to high salinity and waterlogging. The total RNA was extracted using a Qiagen RNeasy Mini Kit (Qiagen, Hilden, Germany) following Lin et al. (2013; 2014). RNA-seq libraries were constructed using a NEBNext Ultra II RNA library preparation kit. Libraries were constructed for the control (no stress), NaCl (10‰, 20‰, and 30‰), and WL (3 h, 6 h, and 9 h of flooding) treatments, with three biological replicates per sample. A total of 16 libraries were sequenced using an Illumina HiSeq4000 platform, obtaining paired-end reads with an average length of 150 bp. After removing library index sequences, the clean reads were mapped to the *K. obovata* genome (GWHACBH00000000.1; Genome Warehouse; https://ngdc.cncb.ac.cn/gwh/jbrowse/index) using Bowtie2 (Langmead and Salzberg, 2012). The raw counts were quantified and normalized following an established analysis pipeline (Lin et al., 2013). Differentially expressed genes (DEGs) were identified using DEseq2 (Love et al., 2014), with a false discovery rate (FDR) < 0.05. The raw sequencing data were deposited in the National Center for Biotechnology Information (NCBI) database under accession numbers PRJNA1051648 (waterlogging) and PRJNA1051781 (high salinity).

### Weighted gene co-expression network analysis

WGCNA was carried out according to an established protocol (Langfelder and Horvath, 2008). Briefly, WGCNA is a method to analyze the gene expression patterns of multiple samples. According to the concept of scale-free network distribution, the correlation coefficients of the expression matrix are weighted so that highly coevolutionary genes can be allocated to the same gene cluster within the whole network. Then, whole genes can be allocated to multiple modules.

### Statistical analysis

Student’s *t*-tests were carried out using SPSS software (v.19.0) to determine statistically significant differences between treatments (*p < 0.05; **p < 0.01).

## Results

### High salinity and waterlogging reduce growth in *K. obovata*

**Figure 1:**
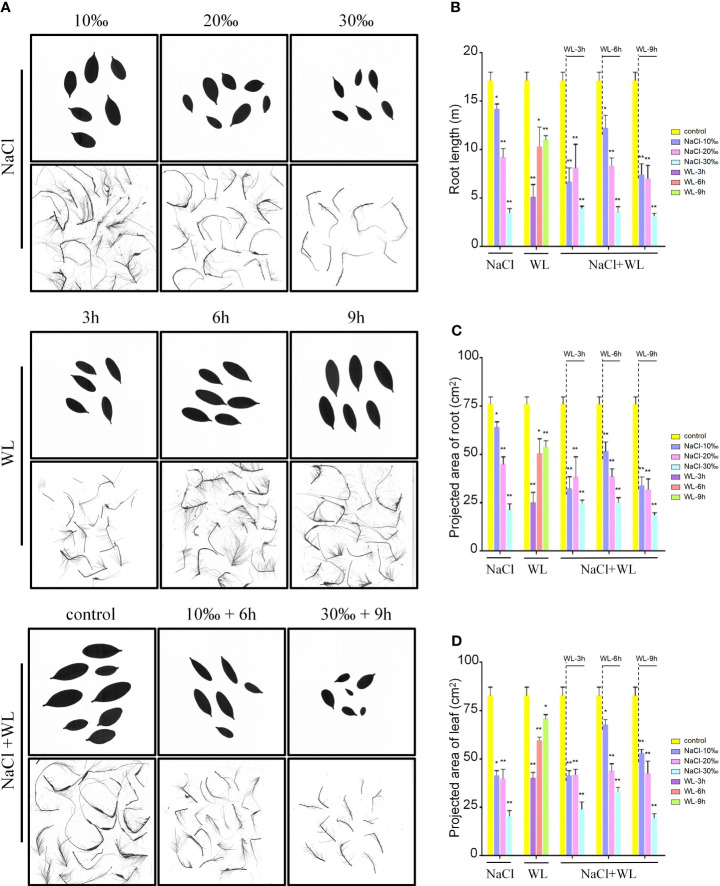
The effects of exposure to high salinity and waterlogging on the growth characteristics of *Kandelia obovata*. Growth performance was analyzed using 6-month-old soil-grown plants. **(A)** Photographs of representative roots and leaves. Bars = 10 cm. **(B–D)** Mean root length (m) **(B)** and mean projected area (cm^2^) of individual roots **(C)** and leaves **(D)**. Error bars represent SEs of three independent experiments. *p < 0.05, **p < 0.01 (Student’s *t*-test).

Exposure to high salinity, waterlogging, and high salinity–waterlogging significantly impacted the root and leaf characteristics of *K. obovata* (**Figure 1**). Compared to control plants, all stress-exposed plants exhibited stunted growth and reduced root length, and the mean projected areas of individual roots and leaves were negatively correlated with salt concentration (**Figure 1**). Notably, growth inhibition gradually weakened with increased waterlogging duration (**Figures 1B–D**). In addition, the magnitude of the negative effects varied with different combinations of salt concentration and waterlogging duration. For example, the most optimal combination was 10‰ NaCl + 6-h waterlogging, while the least optimal was 30‰ NaCl + 9-h waterlogging (**Figures 1B–D**). Exposure to high salinity–waterlogging tended to result in significantly worse growth outcomes than exposure to high salinity or waterlogging separately (**Figure 1**). For example, exposure to high salinity and high salinity–waterlogging decreased root length by 17.2%–79.8% and 28.7%–81.6%, respectively, while exposure to waterlogging alone reduced root length by only 17.2%–39.7% (**Figure 1B**). Similar trends were observed in both surface area and volume (**Supplementary Figure 1**). These results suggest that exposure to high salinity has a more significant impact on the growth of *K. obovata* than exposure to waterlogging.

### High salinity and waterlogging reduce biomass production in *K. obovata*

**Figure 2:**
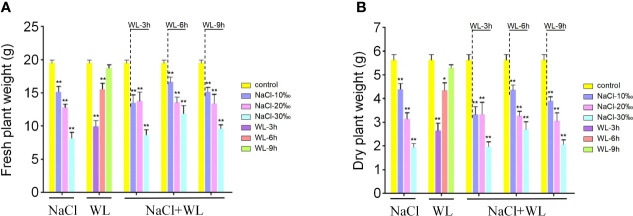
The effects of exposure to high salinity and waterlogging on biomass production in *Kandelia obovata*. **(A, B)** Mean fresh weight (g) **(A)** and mean dry weight (g) **(B)**. Error bars represent SEs of three biological replicates. *p < 0.05, **p < 0.01 (Student’s *t*-test).

The accumulation of biomass in *K. obovata* plants exposed to high salinity and waterlogging was further evaluated. Overall, stress exposure significantly inhibited biomass production (**Figure 2**). Notably, the degree of biomass reduction was positively correlated with salt concentration and negatively correlated with flooding time (**Figure 2**). Biomass production was particularly inhibited in the combined high salinity–waterlogging treatment and also inhibited by high salinity under all waterlogging durations (**Figure 2**). Compared with control plants, exposure to high salinity, waterlogging, and high salinity–waterlogging reduced the fresh weight of *K. obovata* plants by 21.9%–65.4%, 5.9%–52.9%, and 22.3%–64.9%, respectively (**Figure 2A**). The same trend was observed for dry weight (**Figure 2B**). Consistent with the phenotypic results, the optimal combination was found to be 10‰ NaCl + 6-h waterlogging (**Figure 2**). Biomass inhibition was observed in all tested tissues before greenhouse trees were harvested for analysis (**Supplementary Figure 2**). In general, exposure to high salinity resulted in a greater reduction in plant biomass than exposure to waterlogging.

### RNA sequencing results

After 6 months of treatment, RNA-seq was conducted on control plants (no stress) and experimental plants exposed to NaCl (10‰, 20‰, and 30‰) and WL (3 h, 6 h, and 9 h of flooding) to identify key salinity- and waterlogging-responsive genes. Eight RNA-seq libraries (three biological replicates per library) were sequenced for each stress treatment. In total, between 41.23 and 49.81 million clean reads representing between 6,107,823,135 and 7,320,824,121 bp were obtained (**Supplementary Table 1**). The N content of 6,397–11,917 single-ended sequencing reads was 2%–3% of the reading length (**Supplementary Table 2**). The base error rate was 2.95%–3.15%, the Q20 was 95.40%, the Q30 was 88.79%, and the guanine–cytosine (GC) content was between 44.77% and 45.31% (**Supplementary Figure 3** and **Supplementary Table 2**). Pearson’s correlation coefficients were calculated using R and indicated that the three replicates for each treatment exhibited high reproducibility (**Supplementary Figure 4**). Overall, these results suggest that the RNA-seq data were suitable for further analysis.

### Salinity- and waterlogging-responsive genes in *K. obovata*

**Figure 3:**
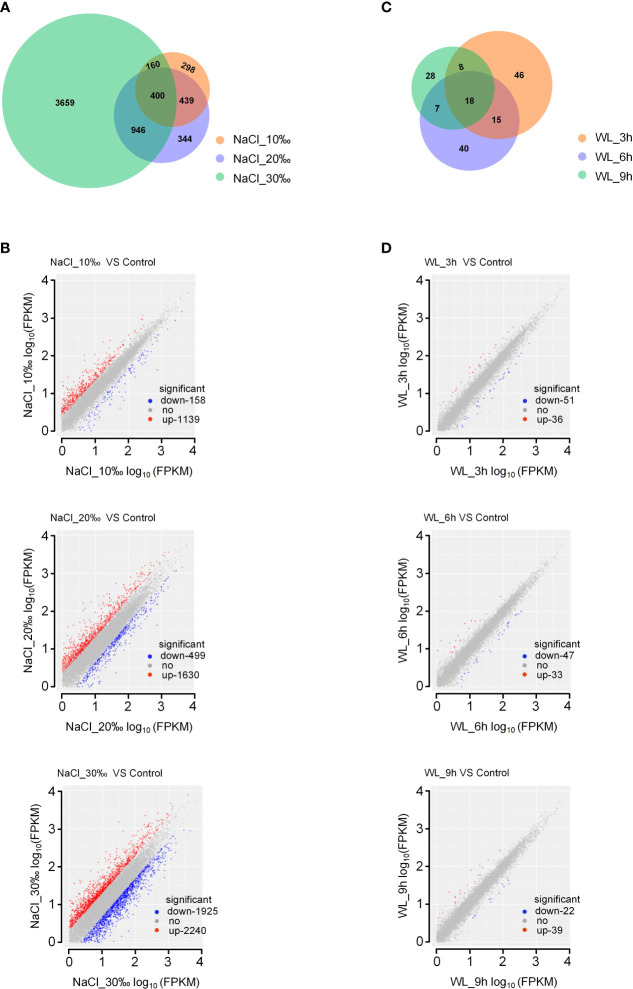
Differentially expressed genes in *Kandelia obovata* exposed to high salinity and waterlogging. **(A, C)** Venn diagrams of salinity-responsive **(A)** and waterlogging-responsive **(C)** differentially expressed genes. **(B, D)** Scatter plots of salinity-responsive **(B)** and waterlogging-responsive **(D)** differentially expressed genes.

In total, we identified 7,591 DEGs across all high-salinity treatments (*p*-value < 0.05; **Figure 3A**; **Supplementary Table 3**). Notably, the number of DEGs increased with increasing salt concentration, with 1,297 DEGs (1,139 upregulated and 158 downregulated) identified under 10‰ NaCl, 2,129 DEGs (1,630 upregulated and 499 downregulated) identified under 20‰ NaCl, and 4,165 DEGs (2,240 upregulated and 1,925 downregulated) identified under 30‰ NaCl (**Figures 3A, B**; **Supplementary Table 3**). Of the 7,591 total identified DEGs, 400 were common to all high-salinity treatments, including genes encoding TFs (**Figure 3A**).

In addition, 228 DEGs were induced in response to waterlogging (*p*-value <0.05; **Figure 3C**; **Supplementary Table 3**). Among these, 87 DEGs (36 upregulated and 51 downregulated) were identified after 3-h waterlogging, 80 DEGs (33 upregulated and 47 downregulated) were identified after 6-h waterlogging, and 61 DEGs (39 upregulated and 22 downregulated) were identified after 9-h waterlogging (**Figure 3D**; **Supplementary Table 3**). Only 18 common DEGs were identified among waterlogging treatments (**Figure 3C**). In accordance with the phenotypic results, exposure to high salinity had a more significant impact on gene expression in *K. obovata* than exposure to waterlogging.

### Principal component analysis

**Figure 4:**
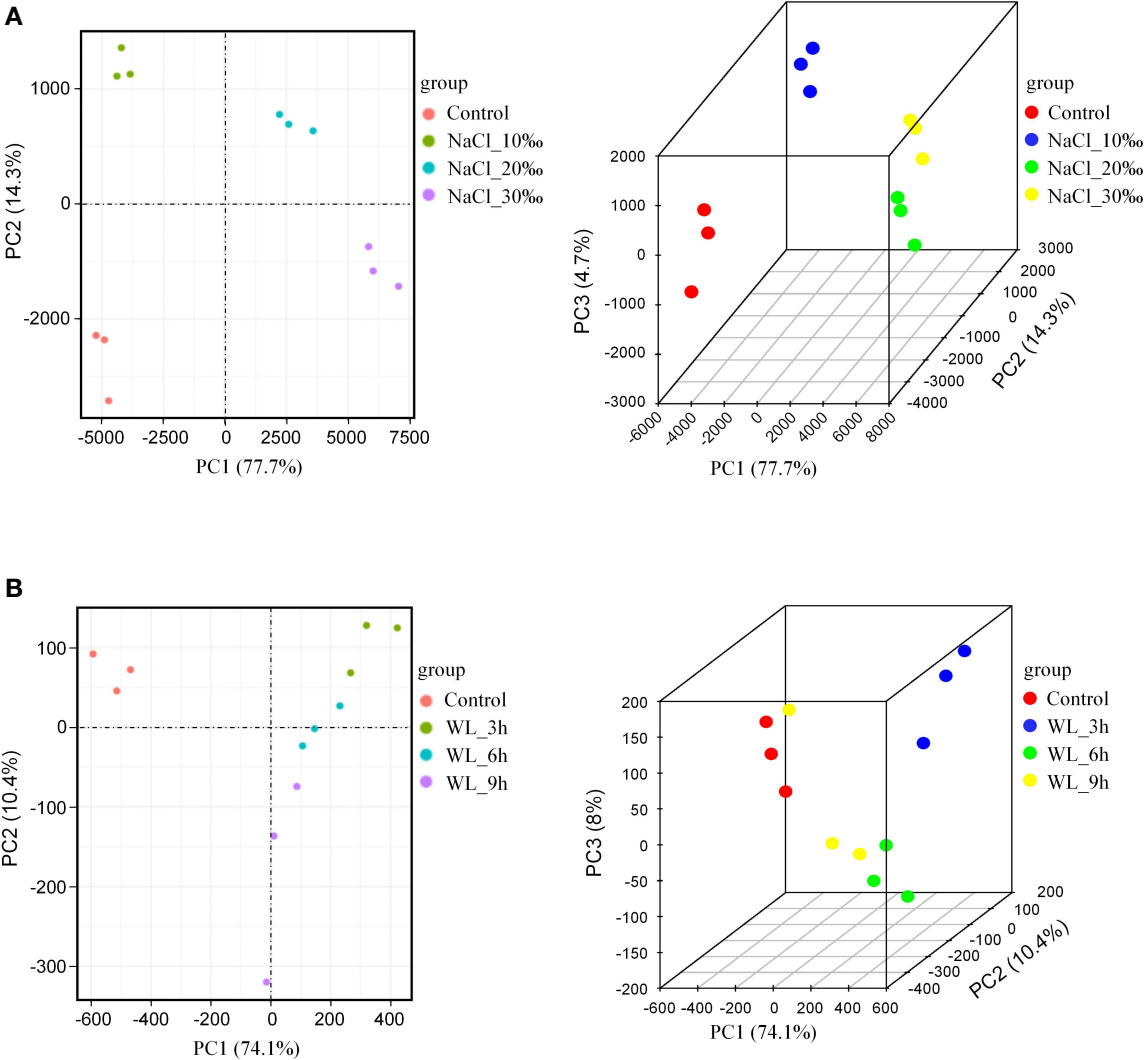
Principal component analysis (PCA). **(A)** PCA of salt stress treatment shown in 2D (left) and 3D (right). **(B)** PCA of waterlogging stress treatment shown in 2D (left) and 3D (right). PC1 is the most influential factor affecting variation, PC2 is the second, and PC3 is the third.

Inter-group differences and intra-group sample duplication were evaluated by principal component analysis (PCA). Overall, genes tended to cluster according to salt concentration, indicating that gene expression was specific to different levels of salinity stress (**Figure 4A**). In addition, the different waterlogging treatments could be separated, albeit with somewhat less specificity (**Figure 4B**). The results of the RNA-seq correlation analysis were similar: different salt concentrations showed good separation, with greater differences in concentration resulting in more pronounced separation (**Supplementary Figure 4A**). However, Pearson’s correlation coefficients were not significantly different between different waterlogging durations (**Supplementary Figure 4B**). Once again, it appears that high salinity has a greater impact on growth than waterlogging in *K. obovata*.

### Weighted gene co-expression network analysis of salinity-responsive gene modules

**Figure 5:**
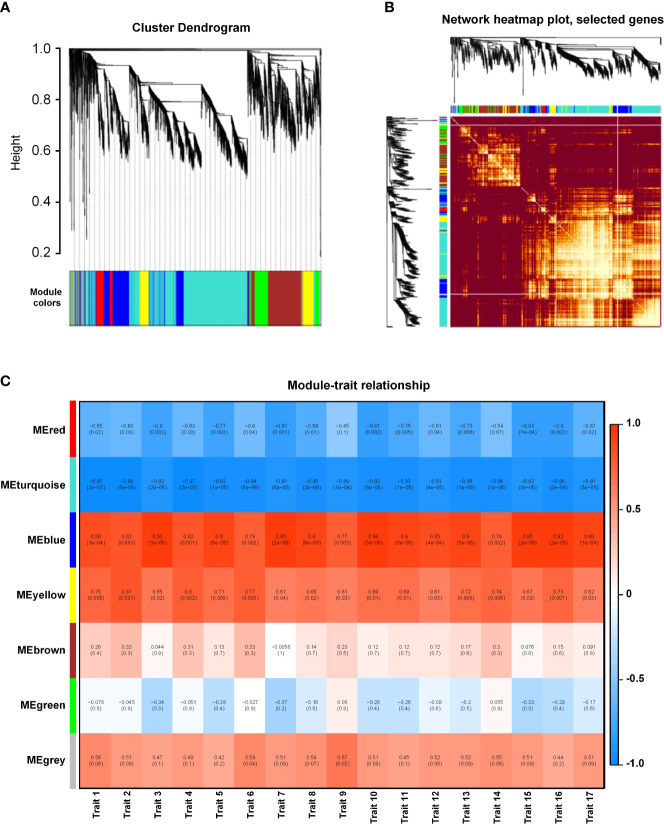
Weighted gene co-expression network analysis (WGCNA) of salinity-responsive gene modules in *Kandelia obovata*. **(A)** Clustering dendrogram with dissimilarity based on topological overlap, together with assigned module colors. **(B)** Heatmap showing Pearson’s correlation among the eigengenes in co-expression gene modules. **(C)** Correlation between modules and traits. Traits 1–17 respectively represent root length, projected root area, projected leaf area, root surface area, leaf surface area, root volume, leaf volume, fresh plant weight, fresh root weight, fresh stem weight, fresh leaf weight, fresh hypocotyl weight, dry plant weight, dry root weight, dry stem weight, dry leaf weight, and dry hypocotyl weight.

We performed a WGCNA of all DEGs to explore the effect of different salt stress concentrations. Overall, the genes were grouped into six co-expression modules according to their pairwise correlation evaluation (**Figures 5A, B**), and each set of highly correlated genes corresponds to a branch on the tree (**Figure 5A**). We observed high topological overlap between genes within the same module. A cluster visualization analysis of salinity-responsive mRNA expression indicated that genes within each module are independent of each other, highlighting the high degree of independence between modules and the relative independence of gene expression within each module (**Figure 5B**). The turquoise module contained the greatest number of eigengenes (2331). With the exception of the gray modules (which contained genes that did not fit into any one module), the red module contained the fewest eigengenes (257) (**Supplementary Figure 5A**). To identify the co-expression modules highly correlated with salinity tolerance, we performed a correlation with the 17 measured phenotypic traits. The blue and yellow modules were significantly associated with all phenotypic characteristics, with correlation coefficients ranging from 0.65 to 0.95 (**Figure 5C**). These results suggest that the eigengenes within these two modules are likely involved in salinity tolerance in *K. obovata*.

### Weighted gene co-expression network analysis of waterlogging-responsive gene modules

**Figure 6:**
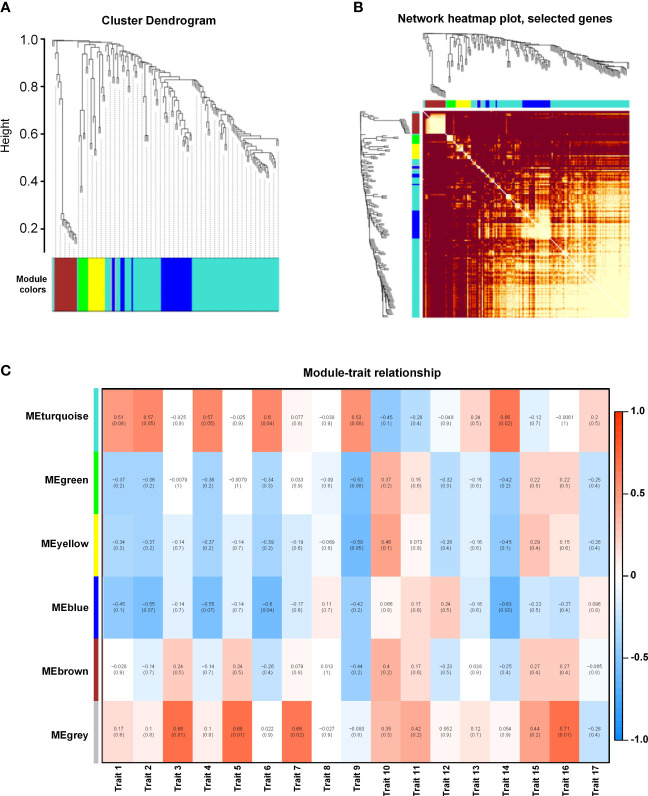
Weighted gene co-expression network analysis (WGCNA) of waterlogging-responsive gene modules in *Kandelia obovata*. **(A)** Clustering dendrogram with dissimilarity based on topological overlap, together with the assigned module colors. **(B)** Heatmap showing Pearson’s correlation among the eigengenes in co-expression gene modules. **(C)** Correlation between modules and traits. Traits 1–17 respectively represent root length, projected root area, projected leaf area, root surface area, leaf surface area, root volume, leaf volume, fresh plant weight, fresh root weight, fresh stem weight, fresh leaf weight, fresh hypocotyl weight, dry plant weight, dry root weight, dry stem weight, dry leaf weight, and dry hypocotyl weight.

We performed a WGCNA of all DEGs to explore the effect of different waterlogging durations. Overall, we grouped the genes into five co-expression modules according to their pairwise correlation evaluation (**Figures 6A, B**). Cluster visualization analysis showed high levels of interdependence between modules and the relative independence of gene expression within each module (**Figure 6B**). The turquoise module contained the greatest number of eigengenes (96), and the green module contained the fewest (7) (**Supplementary Figure 5B**). We performed a correlation with the 17 measured phenotypic traits and found that the turquoise module was highly correlated with trait 7 (root volume) and trait 15 (dry root weight) (**Figure 6C**). These results suggest that the eigengenes within this module are likely involved in waterlogging tolerance in *K. obovata*.

### Functional prediction of salinity- and waterlogging-responsive modules

**Figure 7:**
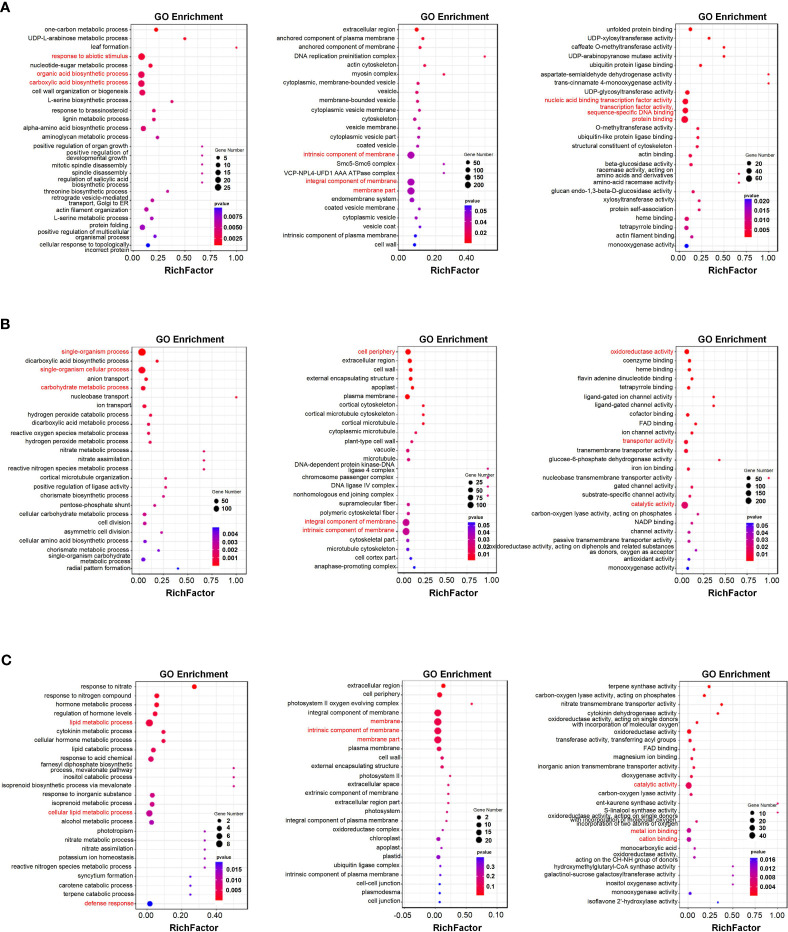
Functional enrichment analyses of key salinity- and waterlogging-responsive modules. **(A, B)** Gene Ontology (GO) enrichment analysis of genes in the salinity-responsive MEblue **(A)** and MEyellow **(B)** modules. **(C)** GO enrichment analysis of genes in the waterlogging-responsive MEturquoise module. Each figure depicts biological pathways (left), cell components (middle), and molecular functions (right). The node size reflects the gene count, and the node color reflects the *p*-value [−log10^(^ *^p^* ^-value)^].

The key modules identified in the WGCNA were subjected to Gene Ontology (GO) functional enrichment analysis. **Figures 7A, B** summarize the GO enrichment analysis results for the salinity-responsive MEblue and MEyellow modules. The MEblue module was the most significantly enriched in the response to abiotic stimulus (GO:0009628), organic acid biosynthetic process (GO:0016053), and carboxylic acid biosynthetic process (GO:0046394) biological processes (BPs); the membrane part (GO:0044425), intrinsic component of membrane (GO:0031224), and integral component of membrane (GO:0016021) cellular components (CCs); and the protein binding (GO:0005515), nucleic acid binding transcription factor activity (GO:0001071), and transcription factor activity, sequence-specific DNA binding (GO:0003700) molecular functions (MFs) (**Figure 7A**). The MEyellow module was the most significantly enriched in the single-organism process (GO:0044699), single-organism cellular process (GO:0044763), and carbohydrate metabolic process (GO:0005975) BPs; the intrinsic component of membrane (GO:0031224), integral component of membrane (GO:0016021), and cell periphery (GO:0071944) CCs; and the catalytic activity (GO:0003824), oxidoreductase activity (GO:0016491), and transporter activity (GO:0005215) MFs (**Figure 7B**). Overall, eigengenes in both modules were related to plant abiotic stress response, indicating that these genes likely play a role in salinity tolerance in *K. obovata*.

**Figure 7C** summarizes the GO enrichment analysis results for the waterlogging-responsive MEturquoise module. The MEturquoise module was the most significantly enriched in the lipid metabolic process (GO:0006629), cellular lipid metabolic process (GO:0044255), and defense response (GO:0006952) BPs; the membrane (GO:0016020), membrane part (GO:0044425), and intrinsic component of membrane (GO:0031224) CCs; and the catalytic activity (GO:0003824), metal ion binding (GO:0046872), and cation binding (GO:0043169) MFs. Overall, eigengenes in the turquoise module were related to plant abiotic stress response, indicating that these genes likely play a role in waterlogging tolerance in *K. obovata*.

**Figure 8:**
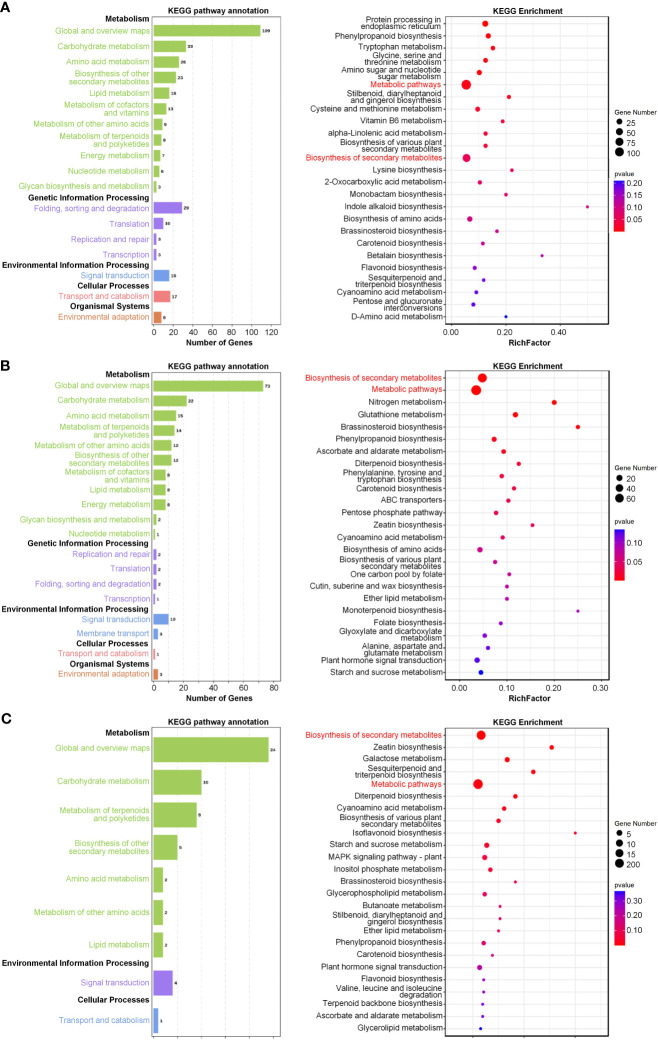
Pathway enrichment analyses of key salinity- and waterlogging-responsive modules. **(A, B)** Kyoto Encyclopedia of Genes and Genomes (KEGG) analysis of genes in the salinity-responsive MEblue **(A)** and MEyellow **(B)** modules. **(C)** KEGG analysis of genes in the waterlogging-responsive MEturquoise module. The node size reflects the gene count, and the node color reflects the *p*-value [−log10^(^ *^p^* ^-value)^].

We further carried out Kyoto Encyclopedia of Genes and Genomes (KEGG) pathway analysis in order to determine the pathways in which the genes in these three modules were involved. Notably, the genes within each of the modules were found to be related to metabolic processes (**Figure 8**). Genes within the salinity-responsive MEblue module were related to metabolic pathways, biosynthesis of secondary metabolites, and phenylpropanoid biosynthesis (**Figure 8A**). Genes within the salinity-responsive MEyellow module were related to metabolic pathways, the biosynthesis of secondary metabolites, and plant hormone signal transduction (**Figure 8B**). Finally, genes within the waterlogging-responsive MEturquoise module were related to metabolic pathways, secondary metabolite biosynthesis, and galactose, starch, and sucrose metabolism (**Figure 8C**).

### Screening of core salinity- and waterlogging-responsive genes

**Figure 9:**
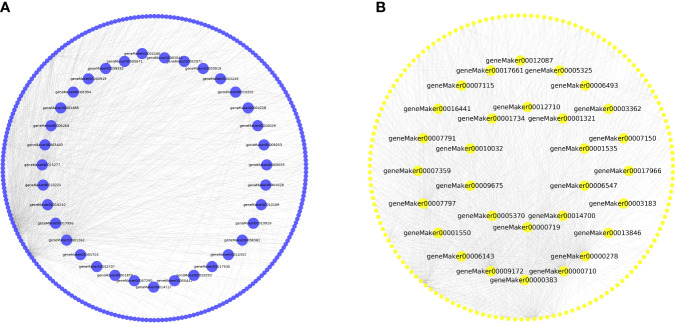
Gene interaction network of hub genes. **(A)** MEblue module. **(B)** MEyellow module.

To identify key genes within the co-expression network, we used Cytoscape to visualize the gene network map (Shannon et al., 2003). The co-expression network in the salinity-responsive MEblue module contained 24 TFs and 251 structural genes (**Figure 9A**, **Supplementary Table 4**). Among these, 35 key genes were found to be related to salt stress, including three TFs: geneMaker00002309, geneMaker00007290, and geneMaker00012453 (**Figure 9A**, **Supplementary Table 4**). The salinity-responsive MEyellow module co-expression network contained 17 TFs and 160 functional genes (**Figure 9B**, **Supplementary Table 4**). Ten genes were found to act as the core of the interaction network, including the TF geneMaker00006547 (**Figure 9B**, **Supplementary Table 4**). The 45 core genes in the MEblue and MEyellow modules related to salt stress were mainly involved in metabolic pathways and the biosynthesis of secondary metabolites (**Supplementary Table 5**). The TFs within these two modules belonged to 12 and 13 families (**Figure 9B**, **Supplementary Table 4**), highlighting the sensitivity of *K. obovata* to salt stress. The homologous *Arabidopsis thaliana* genes encoding these TFs were identified as BBX21 (also known as salt tolerance homolog2 (STH2)), WRKY23, and MYBD. Research indicates that these TFs regulate shoot branching, phytohormone biosynthesis, photomorphogenesis, and abiotic stress response (Ding et al., 2013; Guo and Qin, 2016; Crocco et al., 2018; Xu et al., 2018). Finally, the co-expression network in the waterlogging-responsive MEturquoise module contained 16 genes, which were involved in metabolic pathways and the biosynthesis of secondary metabolite pathway (**Supplementary Table 5**). The only identified TF was geneMaker00001122, which was at the core of the interaction network (**Supplementary Figure 7**, **Supplementary Table 4**).

## Discussion

Usually, the mangrove growth zone is located on the tidal flat above the average sea level. Salinity and waterlogging time are both the main limiting factors for the growth of mangrove plants. The results of literature studies on the impact of waterlogging on the growth of *K. obovata* young individuals are inconsistent. In a salinity environment of 10‰, the optimal waterlogging time for *K. obovata* was 8–12 h per day (Liao et al., 2009). Additionally, it had been found that in a 15‰ salinity environment, the optimal waterlogging time was 4–8 h per day for *K. obovata* (Chen et al., 2005). The reasons for these differences may be due to the different simulated salinities and experimental durations in these studies. The former experiment lasted for 3 months, while the latter lasted for 5 months. Overall, longer waterlogging time inhibited the plant growth of *K. obovata*. In this study, it was found that a longer waterlogging duration led to a decrease in the length, surface area, and volume of roots in *K. obovata*, as well as a decrease in biomass accumulation. Field observation showed that the *K. obovata* plants grew well in an 8‰–25‰ salinity environment (Li, 2000). The growth of *K. obovata* seedlings was inhibited when the salinity was higher than 25‰ in the controlled condition (Liao, 2010). The biomass accumulation of *K. obovata* was lower in the higher-salinity environment in this study. There are reports of differences between the salt tolerance ranges observed in field observations and controlled experiments on mangrove plants. This may be due to the periodic changes in environmental salinity. The periodicity of tides and the supply of freshwater in the field were different from the stability of salinity under controlled conditions. In addition, compared to waterlogging, salinity had a more significant impact on the growth of young *K. obovata* plants, and there was no significant interaction between the two factors, which was consistent with the results of literature studies (Liao, 2010).

Here, we not only measured and examined the root morphological parameters and biomass accumulation under salt and waterlogging stress but also performed RNA-seq and WGCNA to correlate the phenotypic data with gene expression patterns, and we discovered core TFs and hub genes. Overall, waterlogging was found to inhibit the growth of *K. obovata*, which was negatively correlated with waterlogging duration. However, the reason for the low correlation between phenotypic characteristics and transcriptomic results under waterlogging stress remains unclear. The reason may have to do with the fact that our waterlogging stress treatment was not dynamic or perhaps may be related to certain innate adaptive traits of the plants themselves. When stressful environmental conditions remain stable, plants may express stress-related genes early but adapt as the duration increases. On the contrary, we found that certain early responders were restored in later periods. Therefore, we suggest that adaptive traits are the more likely explanation, as the observed phenotypic changes did not significantly alter differential gene expression.

Mangroves respond to high salinity and waterlogging through a complex array of molecular mechanisms. WGCNA can provide valuable insights into convoluted genomic networks by mining data to predict gene clusters linked to certain conditions. We performed WGCNA to search for potential biomarkers of salinity and waterlogging stress in *K. obovata*. Specifically, we performed RNA-seq on *K. obovata* exposed to high salinity and waterlogging and correlated the gene expression levels with phenotypic traits to identify core salinity- and waterlogging-responsive modules (**Figures 5**, **6**). Genes within the salinity-responsive MEblue module were highly correlated with traits 3, 5, 7, 10, 11, 13, 15, and 16 (correlation coefficient ≥ 0.9; **Figure 5**), indicating that these genes were activated or repressed in response to high salinity. In contrast, only traits 6 and 14 were correlated with genes within the waterlogging-responsive MEturquoise module, albeit insignificantly (**Figure 6**), indicating that these genes may be weakly related to flooding stress. According to GO enrichment and KEGG pathway analysis, we found that genes in these key modules were enriched in biological processes related to salinity and waterlogging tolerance, such as response to abiotic stimulus, the organic acid biosynthetic process, the carboxylic acid biosynthetic process, the lignin metabolic process, defense response, the biosynthesis of secondary metabolites, metabolic pathways, phenylpropanoid biosynthesis, and plant hormone signal transduction. Therefore, it appears that genes within the key modules identified by WGCNA may be crucial for salinity and waterlogging tolerance in *K. obovata*.

Secondary metabolites are essential to plant life activities, play a significant part in how plants adapt to their surroundings, and are frequently connected to plants’ indirect defense (Kutchan, 2001; Richter et al., 2015; Alseekh and Fernie, 2018). For instance, UV-B irradiation can raise the amount of flavonoid–secondary metabolites in the fruticose lichen *Cladonia arbuscula* (Buffoni Hall et al., 2002), *Arabidopsis* (Li et al., 1993), and *Camellia sinensis* (Zagoskina et al., 2005). Secondary metabolites as multifunctional compounds were involved in many stress reactions such as salt and heavy metals (Kumar et al., 2012; Lushchak and Mosiichuk, 2012; Yusuf et al., 2012; Wang et al., 2016; Li et al., 2019; Jayaraman et al., 2021). In this study, we identified many salt- and waterlogging-related core genes involved in the biosynthesis of secondary metabolites in *K. obovata*. These results indicate that the secondary metabolic pathway plays an important role in response to salt and waterlogging stress, and it may be involved in the expression of related genes in each stress response process.

Phenylpropanoid is one of the secondary metabolites in plants and contributes to plant development and plant–environment interplay (Dong and Lin, 2021). Lignin is the main branch of the phenylpropyl metabolic pathway. Recent studies revealed that transcriptional upregulation of lignin biosynthesis genes such as C4H, C3H, CAD, F5H, HCT, 4CL, COMT, CCR, and CCoAOMT results in the deposition of lignin, thickening of the secondary cell wall, and enhanced salt and osmotic resistance (Dong and Lin, 2021; Liu et al., 2022). As shown in **Figure 8**, the salt- and waterlogging-response genes were enriched into the phenylpropanoid metabolism pathway, suggesting that phenylpropanoids might play vital roles in *K. obovata* coping with natural harsh conditions. Thus, further research is needed to investigate the contribution of phenylpropanoid metabolism to the high salinity and waterlogging resistance of *K. obovata*.

When plants are stimulated by the environment, the signaling pathway of plant hormones is activated, thus playing an important role in the response to various biological and abiotic stresses. Under salt stress, the expression of ethylene (ET) signaling pathway-related genes was upregulated by the MAPK3 pathway to enhance plant tolerance to salt stress (Shu et al., 2022). The increase in jasmonic acid (JA) synthesis can enhance the salt tolerance of *S. lycopersicum*, *O. sativa*, and *Zea mays* L. (Capiati et al., 2006; Wan and Xin, 2022). Moreover, JA can also participate in the resistance of plants to high temperature, low temperature, drought, and heavy metal stress (MaChado et al., 2015; Ghorbel et al., 2021; Zeng et al., 2022). In addition, abscisic acid (ABA), salicylic acid (SA), gibberellin (GA), indole-3-acetic acid (IAA), and brassinolide (BR) are also widely involved in abiotic stress response in plants (Ali and Baek, 2020). Our research results also found that the salt- and waterlogging-related genes were distributed in hormone signaling pathways, so the response of plant hormones to stressed environments can be used as the direction of subsequent research.

## Conclusion

In this study, we evaluated the effects of high salinity and waterlogging on the growth response and gene expression of the mangrove *K. obovata*. We combined growth indices, microscopic observation, RNA-seq, PCA, and WGCNA to elucidate the molecular mechanisms underlying salinity and waterlogging stress tolerance in this important mangrove species. We found that both growth and biomass accumulation were significantly inhibited by high salinity and waterlogging, although the effects were much more dramatic in response to high salinity. Notably, growth was negatively correlated with salt concentration and positively correlated with waterlogging duration. High-throughput RNA-seq successfully identified 7,591 salinity-responsive genes and 228 waterlogging-responsive genes. WGCNA on gene expression and phenotypic characteristics identified 45 highly correlated core genes related to salt stress and 16 poorly correlated genes related to waterlogging stress. All the core genes are mainly involved in metabolic pathways and the biosynthesis of secondary metabolites. We preliminarily defined the enrichment pathway of the salinity- and waterlogging-response genes in *K. obovata*. We speculate that the low correlation resulted from adaptation to long-term waterlogging stress, which leads to the recovery of gene expression over time. In summary, our study preliminarily analyzed the molecular mechanism of salinity and waterlogging stress tolerance in *K. obovata*. These results will be useful for improving the stress resistance of mangrove forest trees using molecular breeding techniques.

## Data availability statement

The data presented in the study are deposited in the National Center for Biotechnology Information Sequence Read Archive repository, accession number PRJNA1051648, and PRJNA1051781.

## Author contributions

HL: Data curation, Writing – original draft. XA: Conceptualization, Data curation, Formal analysis, Writing – review & editing. XL: Data curation, Formal analysis, Writing – review & editing. SY: Conceptualization, Formal analysis, Writing – review & editing. YL: Investigation, Methodology, Writing – review & editing. XW: Methodology, Resources, Writing – review & editing. XWL: Methodology, Software, Writing – review & editing. QC: Conceptualization, Formal analysis, Funding acquisition, Writing – review & editing. JW: Conceptualization, Data curation, Formal analysis, Funding acquisition, Resources, Writing – review & editing.
