# Supplementary material for: Molecular mechanism of salinity and waterlogging tolerance in mangrove Kandelia obovata
Source: Front Plant Sci. 2024 Feb 7;15:1354249. doi: 10.3389/fpls.2024.1354249 (PMC10879410; doi:10.3389/fpls.2024.1354249)
Supplement: Supplementary file 1 [file DataSheet_1.zip › Supplementary Figures 1-7.docx]

## Frontiers in Plant Science Supporting Information

Article title: Mechanism of Salinity and Waterlogging Tolerance in Mangrove *Kandelia obovata*

Authors: Huizi Liu^1^, Xia An^2^, Xing Liu^1^, Sheng Yang^1^, Yu Liu^1^, Xin Wei^1^, Xiaowen Li^1^, Qiuxia Chen^1*^, Jinwang Wang^1*^

The following Supporting Information is available for this article:

Fig. S1. Individual and interactive effects of salinity and waterlogging on the surface area and volume of roots and leaves.

Fig. S2. Variations of the biomass accumulation of different *K. obovata* tissues.

Fig. S3. GC content of sequencing results.

Fig. S4. RNA sequencing correlation analysis.

Fig. S5. The number of eigengenes in different salinity- and waterlogging-responsive modules.

Fig. S6. Gene Ontology (GO) enrichment analysis.

Fig. S7. Gene interaction network of hub genes.

Table S1. List of salt and waterlogging stress treatments in *Kandelia obovata.*

Table S2. RNA sequencing reads and sequence coverage .

Table S3. Salinity- and waterlogging-responsive differentially expressed genes in *Kandelia obovata*.

Table S4. Genes in the co-expression networks of key salinity- and waterlogging-responsive modules.

Table S5. List of KEGG pathways of core genes in different modules.


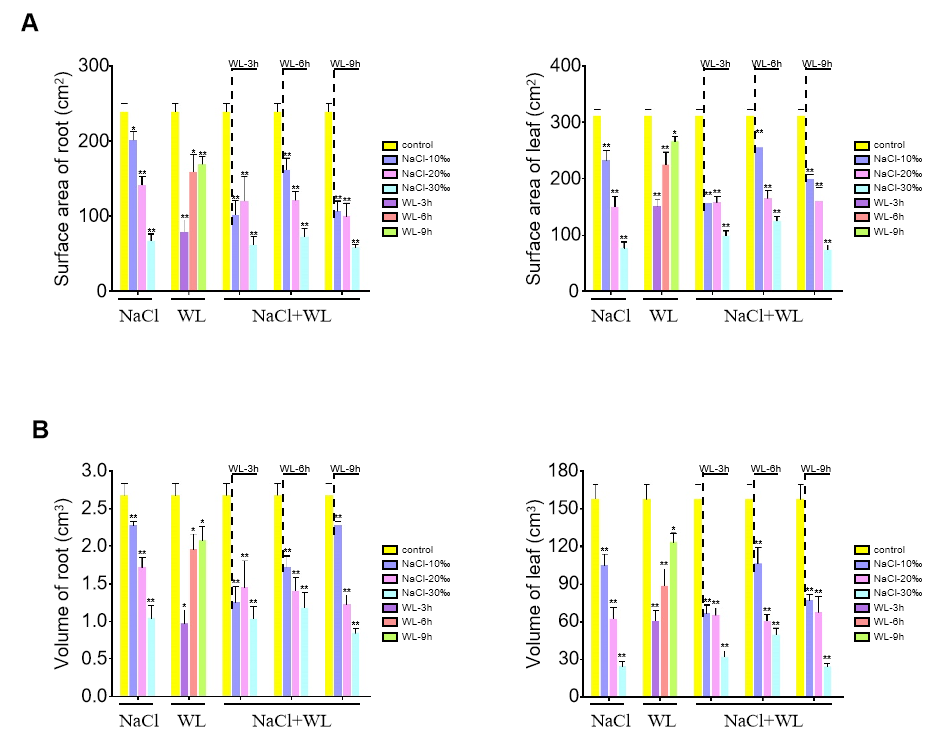


**Fig. S1 Individual and interactive effects of salinity and waterlogging on the surface area and volume of roots and leaves.** Individual and interactive effects of salinity and waterlogging on the surface area and volume of roots and leaves. (A, B) Statistical analysis of mean surface area (cm2; A) and mean volume (cm3; B) of individual roots and leaves of *K. obovata* plants with different stress treatments. Error bars represent SE values from three biological replicates, and asterisk indicates significant difference between each line of the stress treated plant and control plants by Student’s *t*-test (**P* < 0.05, ***P* < 0.01 ).


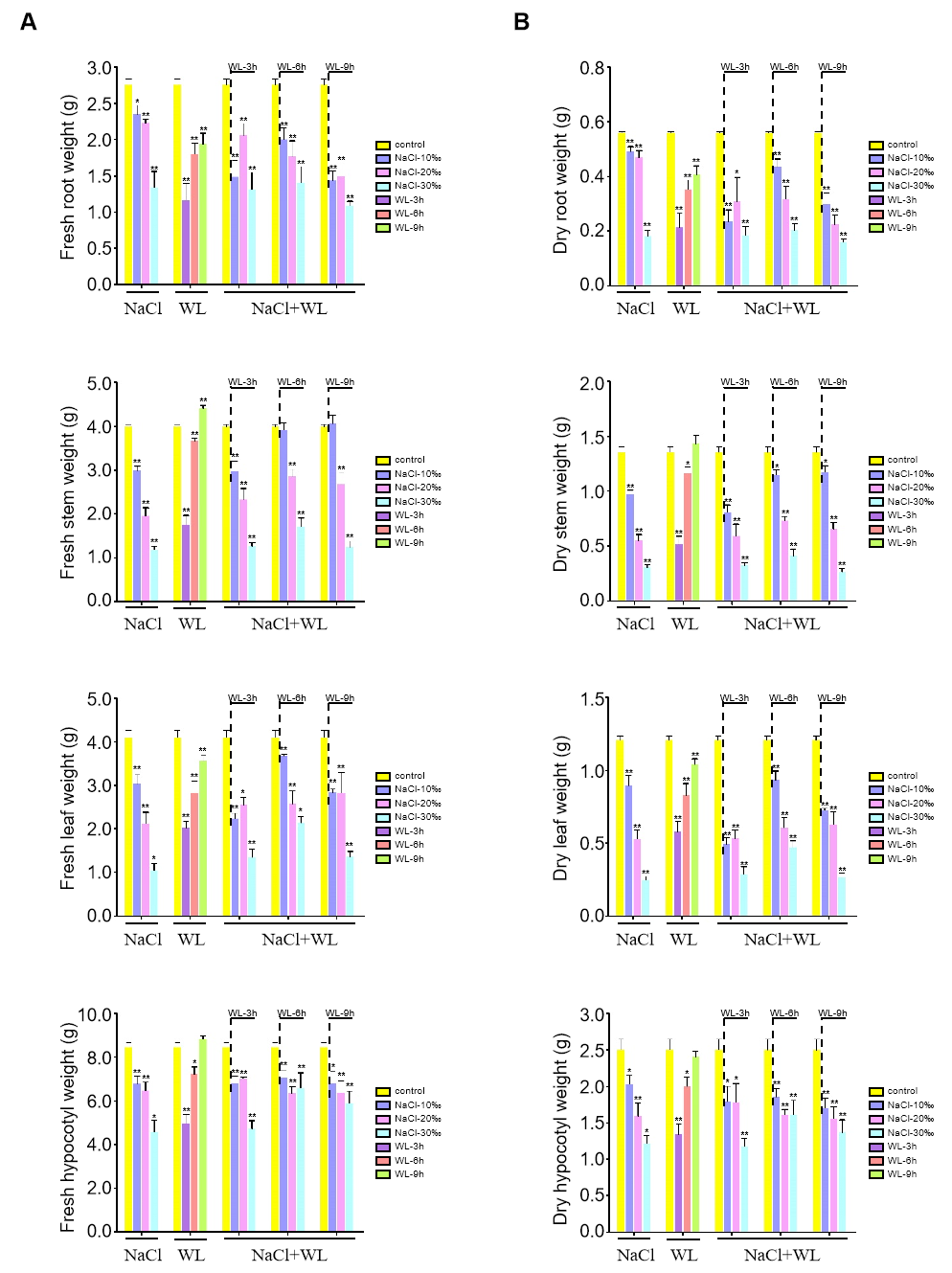


**Fig. S2 Variations in the biomass accumulation of different *K. obovata* tissues. (A, B)** Statistical analysis of fresh weight (g; **A**) and dry weight (g; **B**) of different tissues. Error bars represent SE values from three biological replicates. (**P* < 0.05, ***P* < 0.01, Student’s *t*-test ).


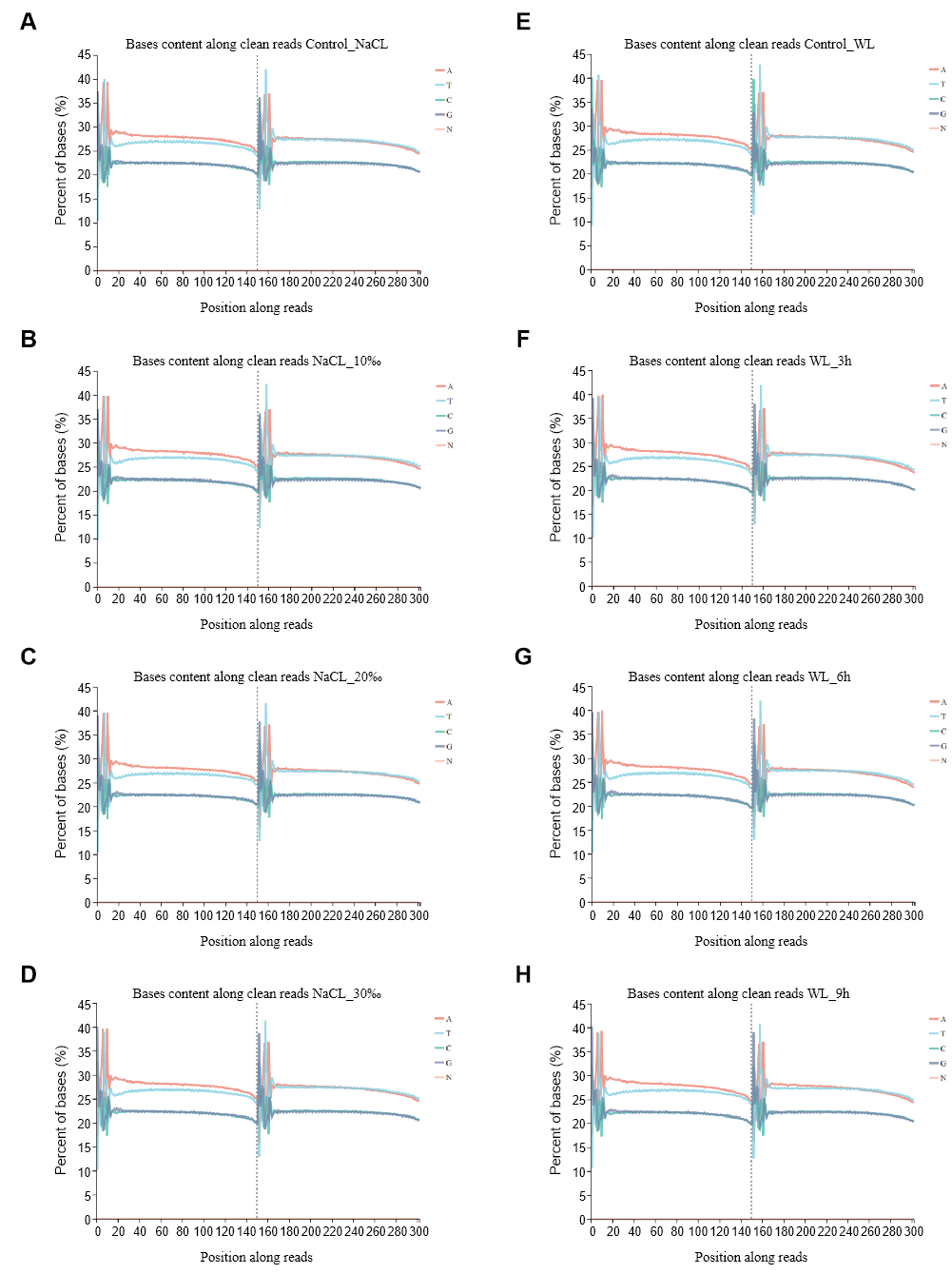


**Fig. S3 GC content of sequencing results. (A-B)** *K. obovata* plants were treated with various concentrations of NaCl three repeated experiments GC content statistics of sequencing results. (**E-H)** *K. obovata* plants were treated with different time of waterlogging three repeated experiments GC content statistics of sequencing results. The x-coordinate is the base position of reads, and the y-coordinate is the percentage of the five base types of ATGCN.


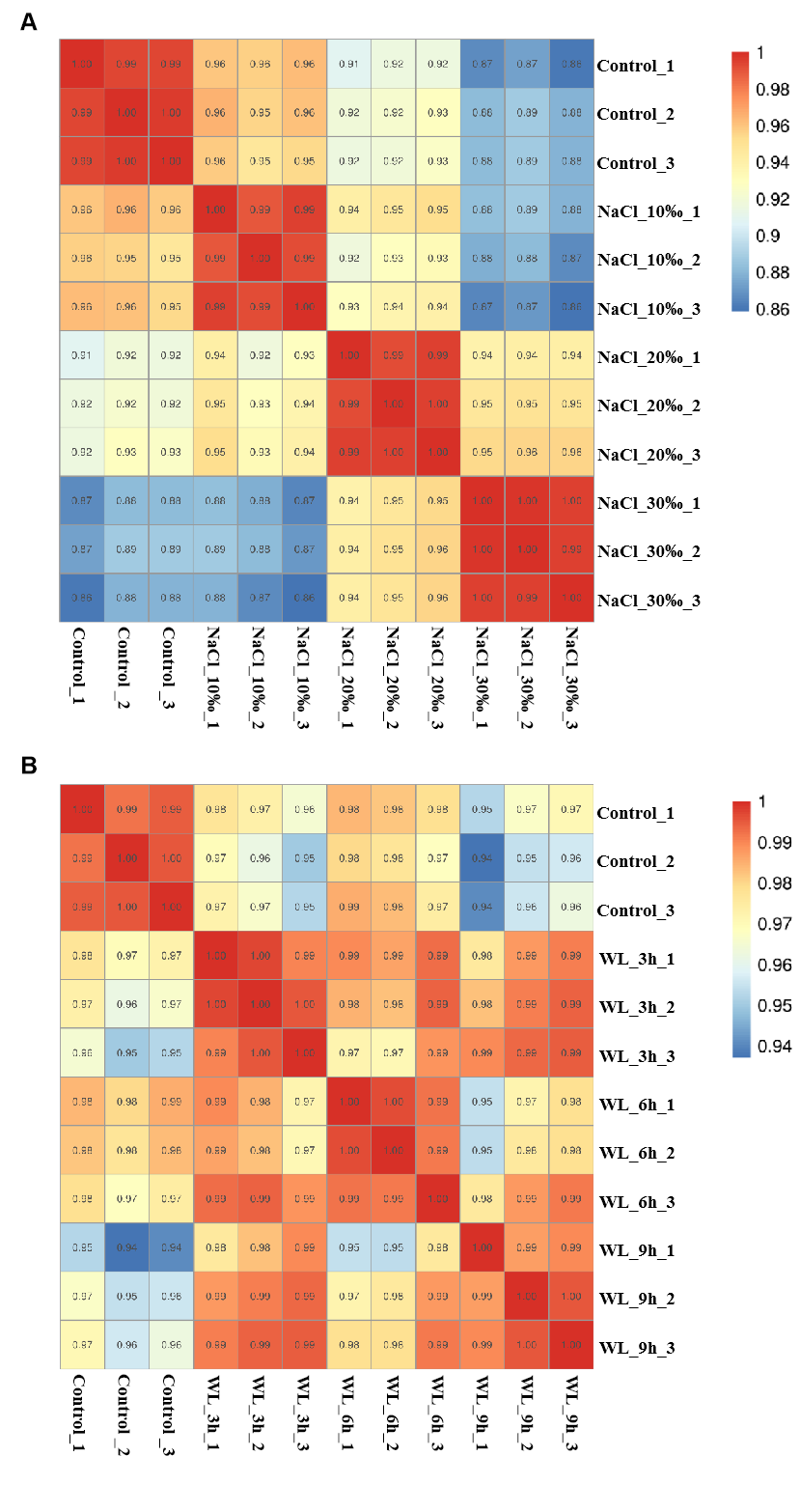


**Fig. S4 RNA** **sequencing correlation analysis. (A)** Correlation analysis of data with various concentrations of NaCl stress treatments. **(B)** Correlation analysis of data with different time of waterlogging stress treatments. Row and column are sample names, and the intersection of row and column is correlation coefficient between samples (the Pearson correlation coefficient, R). The larger the value, the stronger the correlation.


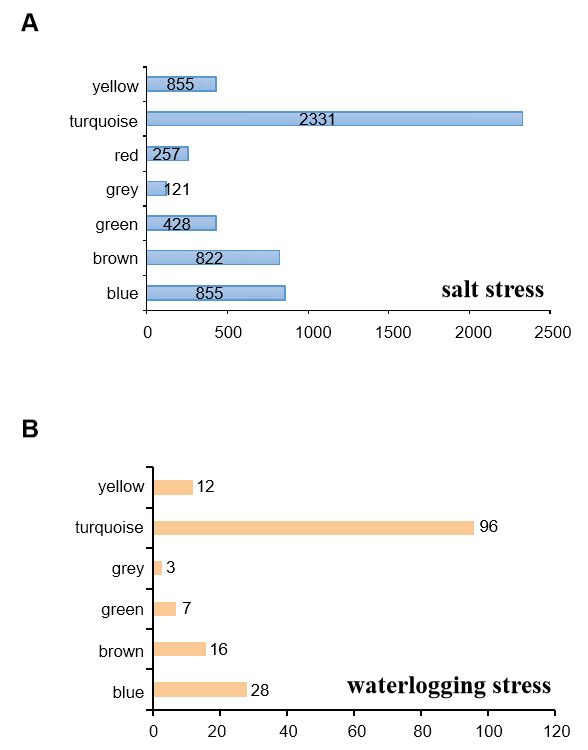


Fig. S5 The number of eigengenes in different modules of salinity- and waterlogging-responsive modules. Grey modules represent genes that don't fit into any one module.


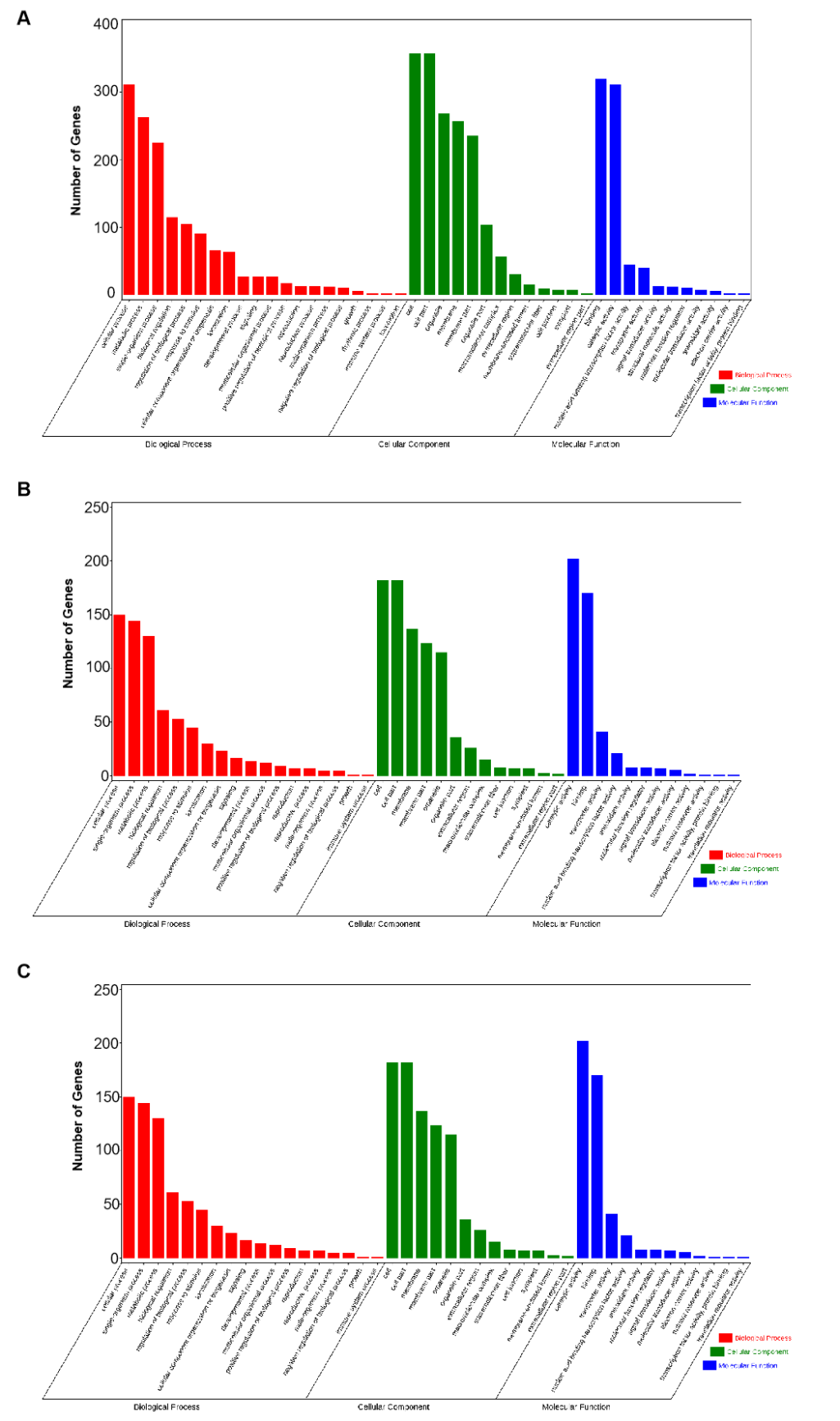


**Fig. S6** **Gene Ontology (GO) enrichment analysis. (A)** Most enriched GO terms in the MEblue module under salt stress. **(B)** Most enriched GO terms in the MEyellow module under salt stress. **(C)** Most enriched GO terms in the MEturquoise module under waterlogging stress.


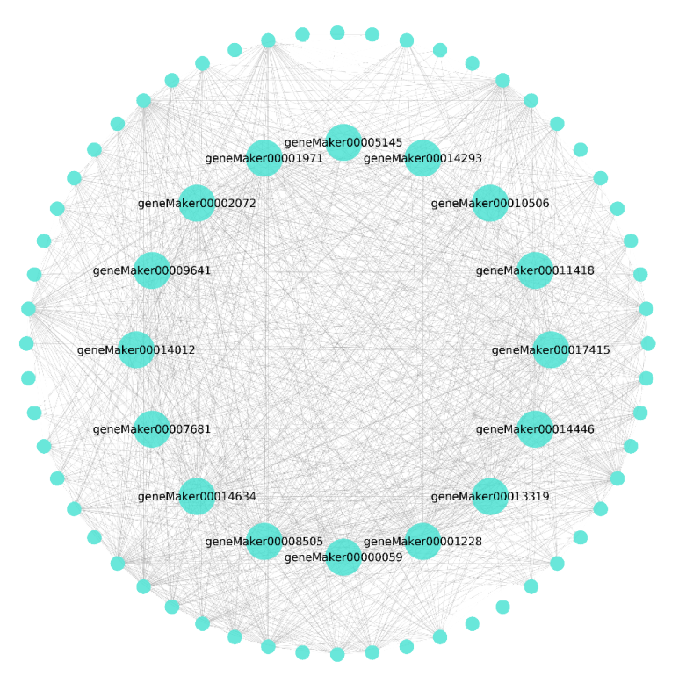


Fig. S7 Gene interaction network of hub genes.
